# Spatial swarm segregation and reproductive isolation between the molecular forms of *Anopheles gambiae*

**DOI:** 10.1098/rspb.2009.1167

**Published:** 2009-09-04

**Authors:** Abdoulaye Diabaté, Adama Dao, Alpha S. Yaro, Abdoulaye Adamou, Rodrigo Gonzalez, Nicholas C. Manoukis, Sékou F. Traoré, Robert W. Gwadz, Tovi Lehmann

**Affiliations:** 1 Laboratory of Malaria and Vector Research, National Institute of Allergy and Infectious Diseases, National Institutes of Health, 20852 Rockville, MD, USA; 2 Malaria Research and Training Center, Bamako, Mali

**Keywords:** molecular forms, *Anopheles gambiae*, reproductive isolation, swarms

## Abstract

*Anopheles gambiae*, the major malaria vector in Africa, can be divided into two subgroups based on genetic and ecological criteria. These two subgroups, termed the M and S molecular forms, are believed to be incipient species. Although they display differences in the ecological niches they occupy in the field, they are often sympatric and readily hybridize in the laboratory to produce viable and fertile offspring. Evidence for assortative mating in the field was recently reported, but the underlying mechanisms awaited discovery. We studied swarming behaviour of the molecular forms and investigated the role of swarm segregation in mediating assortative mating. Molecular identification of 1145 males collected from 68 swarms in Donéguébougou, Mali, over 2 years revealed a strict pattern of spatial segregation, resulting in almost exclusively monotypic swarms with respect to molecular form. We found evidence of clustering of swarms composed of individuals of a single molecular form within the village. Tethered M and S females were introduced into natural swarms of the M form to verify the existence of possible mate recognition operating within-swarm. Both M and S females were inseminated regardless of their form under these conditions, suggesting no within-mate recognition. We argue that our results provide evidence that swarm spatial segregation strongly contributes to reproductive isolation between the molecular forms in Mali. However this does not exclude the possibility of additional mate recognition operating across the range distribution of the forms. We discuss the importance of spatial segregation in the context of possible geographic variation in mechanisms of reproductive isolation.

## 1. Introduction

Ecologically based divergent selection is a process in which different phenotypes are favoured by different environments. If the variation between phenotypes has a genetic basis, different environments will favour different alleles, resulting in ecologically based divergent evolution. Ultimately, reproductive isolation evolves as a consequence of this selection. The process is known as ecological speciation and it might occur in allopatry or in sympatry (Schluter 2001). Rundle and Nosil (2005) separated ecological speciation into three components: an ecological base of divergent selection, a mechanism of reproductive isolation, and a linkage between them. Recent results have revealed that divergent selection between the molecular forms of *Anopheles gambiae* is mediated by predation pressure (Diabaté *et al.* 2008), in accordance with the first component defined by Rundle and Nosil. Here, we investigate the second component, i.e. the mechanisms of reproductive isolation that restrict gene flow between the forms.

*An. gambiae*, the major malaria vector in Africa, is undergoing speciation (Coluzzi *et al.* 2002; della Torre *et al.* 2002). Early studies based on chromosomal inversions of *An. gambiae* in West Africa found five partially isolated populations based on combinations of paracentric inversions on the right arm of chromosome 2. These were named Forest, Savanna, Bamako, Mopti and Bissau chromosomal forms (Bryan *et al.* 1982; Coluzzi *et al.* 1979, 1985; Touré *et al.* 1998). The chromosomal forms exhibit different degrees of gene flow between them, and their spatial and seasonal distribution indicates that they are adapted to different niches.

The distribution range of the chromosomal forms overlaps extensively, except in the semi-desert belt of West Africa, where the Mopti chromosomal form occurs exclusively (Touré *et al.* 1998; Lehmann & Diabaté 2008). The Forest chromosomal form is found in the humid forest belt of West and Central Africa. The Bamako chromosomal form is restricted to the upper Niger river basin and is associated with laterite rock pools as its main larval habitat (Touré *et al.* 1998; Manoukis *et al.* 2008; Sogoba *et al.* 2008).

Subsequent studies revealed two ‘molecular’ forms (M and S) characterized by fixed nucleotide differences in the intergenic spacer of the ribosomal DNA (della Torre *et al.* 2001; Favia *et al.* 2001). The relationship between the molecular and chromosomal forms is complex and depends on geography. The M-form genotype is associated with the chromosomal forms Mopti, Savanna, Forest and Bissau, whereas the S genotype is associated with the chromosomal forms Savanna, Bamako and Forest. In Mali and Burkina Faso, the M form strictly corresponds to Mopti and the S form strictly corresponds to Savanna and Bamako chromosomal forms (della Torre *et al.* 2001). The reproductive isolation between the molecular forms is independent of their chromosomal constitution (Wondji *et al.* 2002). Therefore, chromosome inversions are not linked to the mate recognition system, whereas they are believed to contain genes conferring ecotypic adaptations (Coluzzi *et al.* 2002; della Torre *et al.* 2005).

Typically the S form peaks in the rainy season, exploiting rain-dependent puddles as larval sites, whereas the M form predominates in more arid conditions and in association with irrigated sites such as rice fields (Diabaté *et al.* 2002, 2003, 2004; della Torre *et al.* 2005). Genetic differentiation between the molecular forms is high only in two or three tiny genomic areas named the ‘speciation islands’ (representing less than 1% of the total genome) with low or no differentiation found across most of the genome (Gentile *et al.* 2001; Mukabayire *et al.* 2001; Wondji *et al.* 2002; Lehmann *et al.* 2003; Stump *et al.* 2005; Turner *et al.* 2005; Turner & Hahn 2007). The absence of differentiation across most of the genome is probably due to ongoing gene flow between the molecular forms that continues to homogenize regions of the genome not directly involved in the speciation process (della Torre *et al.* 2002). The rate of natural hybridization between the molecular forms is below 1 per cent (della Torre *et al.* 2001; Wondji *et al.* 2005), although 7 to 20 per cent hybridization was found in restricted locations in Gambia and Guinea-Bissau (Caputo *et al.* 2008; Oliveira *et al.* 2008). Whether this deficit of hybrids reflects hybrid inferiority in the field is not known, but laboratory studies have found no evidence for reduced fitness in hybrids (Diabaté *et al.* 2007). Strong assortative mating between the molecular forms in the field has been described (Tripet *et al.* 2001), but its underlying mechanisms are not known.

*An. gambiae* mates in flight at specific mating stations, and very often over specific landmarks known as swarm markers (Downes 1969; Charlwood *et al.* 2002; Yuval 2006). The swarms are composed of males; females typically approach a swarm, acquire a mate and leave *in copula*. Insects use a variety of stimuli to bring males and females together for mating, including pheromones, visual signals and sound signals, which can operate over long and short ranges (Clements 1999). The way the sexes are attracted to each other may contribute to the specific mate recognition systems, which facilitate species identification and prevent hybridization (Clements 1999). The hypothesis that flight-tone is used for differential mate recognition was not supported by experiments in the laboratory (Tripet *et al.* 2004). Additionally, a recent study using a mark–release experiment of M and S forms in natural houses (absence of swarm markers) found no evidence for assortative mating when mating occurs indoors (Dao *et al.* 2008), suggesting that chemical and sound cues are not involved, at least under these conditions.

Studies on mate recognition between the molecular forms and especially the absence of hybrids and the evidence for assortative mating lead us to hypothesize that reproductive isolation between the molecular forms is associated with mating swarms. In a previous study of swarm composition in Burkina Faso, we found that swarm composition was not random and that the frequency of mixed swarms was far smaller than expected by chance (Diabaté *et al.* 2006), suggesting that swarm segregation contributes to reproductive isolation. However, inference based on that study was limited because we only found swarms of S forms exclusively or mixed swarms, but no swarms of the M form, possibly because of a low abundance of M males (3.2%) at that location and time. Here, we address this hypothesis by further evaluating the contribution of spatial swarm segregation to reproductive isolation between the molecular forms. We show that, in Mali, segregation of swarms is an important mechanism that restricts gene flow between the molecular forms.

## 2. Material and methods

### (a) Study area

A study on swarming behaviour of the molecular forms of *An. gambiae* was conducted in August and September 2006 and 2007 in Donéguébougou, Mali (12° 48′ 38″ N; 7° 59′ 5″ W), located 29 km northeast of Bamako on the edge of a temporary stream surrounded by hills with a small rice cultivation area. During the wet season (1998), *An. gambiae* ss. population in this village comprised 11 to 30 per cent of the Bamako chromosomal form, 4 to 44 per cent of the Savanna form and 33 to 63 per cent of the Mopti form (Touré *et al.* 1998).

### (b) Swarm composition

A survey of swarms was undertaken by trained observers in Donéguébougou, starting at sunset and looking towards the lightest part of the sky from 0.5 to 4 m above the ground. Once located, swarms were collected using an insect net. Mosquitoes were aspirated into cups, killed with chloroform, identified and kept in 80 per cent ethanol in 1.5 ml tubes. The location of the swarm, time of collection, landmark and height above ground were recorded. Observations were made on 19 swarm sites spread throughout the entire village, where swarms were observed forming every evening. Samples were taken from swarms that formed in the same locations over several evenings. Swarm locations were mapped using a global positioning system (GPS) with measurements of latitude and longitude accurate to within 2 m. Collected specimens were identified by polymerase chain reaction (PCR) to the level of species and molecular forms (Fanello *et al.* 2002), and swarms of the S form were subsequently identified with respect to whether they were of the Bamako or Savanna chromosomal forms (Coulibaly *et al.* 2007). Mating pairs were also collected as they fell or flew out of swarms in the 2007 survey. Males and females from these pairs were subsequently identified to species and molecular forms (Fanello *et al.* 2002).

### (c) Indoor resting composition

Pyrethrum spray collection was performed indoors throughout the village to estimate the relative frequency of the different molecular forms. The collection was done in September the day after the last swarm collection to avoid affecting swarm compositions with the pyrethrum spray. To ascertain that the pattern of swarm distribution across the village was not a by-product of spatial distribution of the forms within the village, 2–4 houses, located within 10 m of each swarming site, were selected for indoor collections. All specimens were identified, preserved and subsequently identified to species/molecular form as described above.

### (d) Form recognition within swarm: tethered females experiment

The experiment was conducted in the village of Sokourani, located in a large ricefield area in the district of Niono in northeast Mali (see details in Sogoba *et al.* 2007). The rice irrigation area is occupied exclusively by the M form of *An. gambiae*. Virgin females were produced in the laboratory from egg batches of wild-caught blood-fed and gravid females collected in Donéguébougou. Three- to five-day-old F1 virgin females of one or the other form were individually tethered by gluing a fine line (50 cm long) to the scutum (dorsal face of the thorax), which was tied onto a 2-m pole. After confirming the flying ability of the tethered female, she was introduced into a natural swarm for 5 min. Pairing between the tethered female and a male from the swarm was noted and subsequently the female was dissected to determine if mating was successful (presence of sperm in her spermatheca). The same experiment was also performed in Donéguébougou, but, in contrast to Sokourani, pairing occurred rarely and the number of females inseminated (one) did not allow further interpretation. Hence, only the data collected in Sokourani is presented. The low pairing in Donéguébougou, as opposed to Sokourani, is probably due to the small size of the swarms. During the experiment we noted that the rate of pairing was higher in large swarms than in small swarms. Swarm size in Sokourani ranged from 100–1000 males, whereas the size in Donéguébougou rarely reached or exceeded 100 males.

## 3. Results

### (a) Swarm observations and collections

Swarms began to form 2–5 min after sunset with one or two males observed in zigzag flight, which were then joined by other males, and lasted for 20–40 min. Swarms remained stationary, flying within a 1.5 m radius of an imaginary centre throughout their duration. Swarm height ranged from 0.5 to 3 m above ground, although sometimes they reached up to 4 m for short intervals. Swarms were observed at the same sites repeatedly. Swarms that were observed in the same site on different days were treated as distinct swarms.

A total of 1145 males were collected from 68 swarms (19 sites) from Donéguébougou between August and September in 2006 and the same period in 2007, when both forms coexisted in that village. During swarm collection, the S form comprised 68.30 per cent, the M form 31.61 per cent and *Anopheles arabiensis* 0.09 per cent of the total. Sample size per swarm varied from 5 to 74 males (median = 13). In 2006, 99.02 per cent (203/205 from 13 swarms) of the S specimens were of the Savanna chromosomal form, and the remainder were the Bamako chromosomal form. In 2007, 100 per cent (154/154 from 11 swarms) were of the Savanna chromosomal form.

### (b) Within-swarm form composition

**Figure 1.**
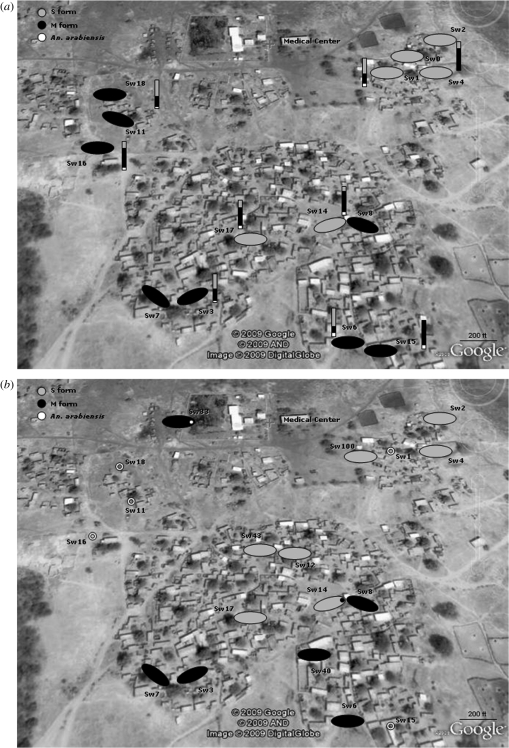
(*a*) Spatial segregation of swarms of the molecular forms (shaded ovals) and indoor composition of the molecular forms collected in the vicinity of the swarms (vertical bars) in 2006. With the exceptions of swarms 0, 11, 16 and 18, all swarms were sampled more than once (2–8 evenings) at the same site. Swarm sizes ranged from 5 to 74. (*b*) Spatial segregation of swarms of the molecular forms (shaded ovals) in 2007. Locations of swarms 1, 11, 15, 16 and 18 are seen on the map, but these swarms were not sampled in 2007. Swarm 33 and swarm 14 are mixed swarms respectively of the M form and *An. arabiensis*, and of the S and M forms.

Swarms were sampled when swarm size was near its peak, between 10 and 20 min after sunset. Swarms usually appeared in the same location every evening. In 2006, identification of 901 males from 47 swarms revealed complete swarm segregation, with every swarm being composed exclusively of either M or S males (figure 1*a*). Three swarms (swarms 1, 2 and 17; figure 1*a*) were sampled three times in the same evening (2 min apart) to assess temporal change in male composition. Overall, 29, 23 and 74 specimens, respectively, were sampled from these swarms (sample size range per time point 2–30), and composition remained 100 per cent of the S form. The composition of all swarm sites sampled at different dates remained unchanged except for one swarm (swarm 17), which consisted exclusively of S males on four evenings (sample size range: 23–74 specimens), but consisted of M males on one evening (sample size, 13 specimens; figure 1*a*).

To further test this pattern, swarm sampling was also performed in the same period in 2007. A total of 244 males were sampled from 21 swarms. Eight of the 13 swarm sites located in 2007 were from the same sites identified in 2006 (swarms 2, 3, 4, 6, 7, 8, 14 and 17). A similar pattern of swarm segregation was observed in 2007, with 20 pure-form swarms and one mixed swarm (figure 1*b*). The sample from swarm 14 had two of the M form and 22 of the S form. The sample from swarm 33 had 14 of the M form and one *An. arabiensis*.

A total of 27 mating couples were collected. Five couples were collected from two M swarms and 22 couples were collected from six S swarms. All couples were homogeneously paired (male and female being of the same molecular form) and were of the same form as the males from the swarms from which they were collected.

### (c) Distribution of the molecular forms resting inside houses

In 2006 and 2007 indoor collections resulted in 394 and 169 *An. gambiae* specimens, respectively (45% males and 55% females). *An. arabiensis* represented 10 per cent of the total collection in 2006 and 2 per cent in 2007. Considering only the molecular forms, the rarer form in 2006 was the S form (47%), whereas in 2007 it was the M form (30%). Importantly, *An. arabiensis* and the molecular forms of *An. gambiae* were spread over the village and found co-inhabiting houses in the vicinity of the different swarms (figure 1*a*). The S molecular form was predominantly of the Savannah chromosomal form (97.6% in 2006, *n* = 167; 100% in 2007, *n* = 116).

Based on the indoor composition of the molecular forms and the number of observed swarms in 2006 and 2007, the expected frequency of mixed swarms was calculated for both years under the assumption of random mixing (no spatial segregation). This expected number was found to be substantially higher than the number of mixed swarms observed (*p* < 0.0001; table 1) both in 2006 and 2007, suggesting a strong segregation in the swarming behaviour of the two forms (table 1).

**Table 1.** Observed and expected number of mixed swarms.

	indoor composition		swarm composition mix / total			
year	*n*^b^	M//S (%)	observed^c^	*n*	expected^d^	*p*^d^
Aug–Sep 2006	394	54//46	0 / 46	901	45.7	<0.0001
Aug–Sep 2007^a^	169	30//70	1 / 21	243	19.7	<0.0001

^a^A single collection was obtained in 2006, 2 weeks after collection of the first swarm, coinciding with the collection of the last swarms. The two collections in 2007 (1–2 September and 13–14 September) were pooled because there was no significant difference in form composition between them (χ^2^ = 1.8208; d.f. = 1; *p* > 0.17).

^b^Total number of mosquitoes collected. Indoor samples include males and females pooled because there was no significant difference between them (*p* > 0.1). Swarm samples consisted of males only.

^c^The number of mixed swarms of the total number of swarms sampled.

^d^Expected number of mixed swarms based on binomial samples drawn from a population with corresponding indoor form composition. Each sample represents a swarm and is of the same sample size as that swarm. Ten thousand simulated sets of swarm samples, each representing the same number of swarms (and the same number of mosquitoes from each swarm) as the actual collection of swarms, were used to enumerate the mixed swarms expected. A mixed sample has at least one member of each swarm (without regard to degree of mixing).

### (d) Swarm markers

**Figure 2.**
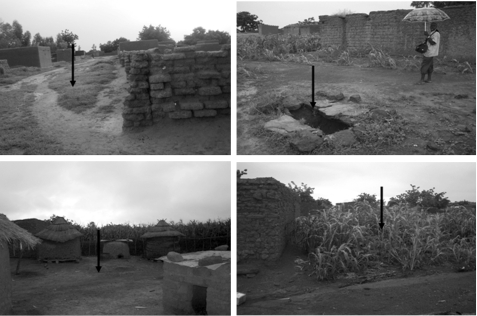
Pictures of representative swarm markers. The arrow indicates the exact placement of the swarm in each site.

**Figure 3.**
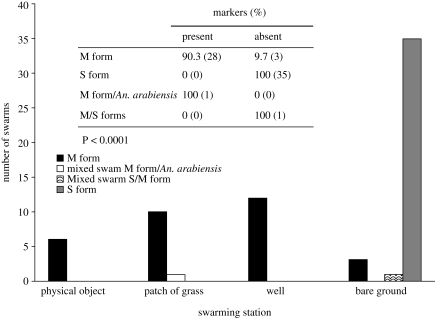
Association between landmarks and swarm of the molecular forms. The M forms swarm above areas of contrast on the landscape, whereas the S form uses no such contrast (table incorporated in figure). The figure gives a brief description of the swarming sites on the *x*-axis.

To understand the role of ground markers in swarm site selection by the molecular forms, all swarm sites were characterized (figure 2). All swarms of the S form were collected over bare ground, whereas the M form was strongly associated with markers consisting of contrasting dark/light pattern, such as the intersection of vegetation (dark) and footpath (light), a water well (dark) surrounded by bare ground (light), and a physical object such as a donkey cart, a chicken house, or a wall on a lighter background (figure 3). Although one M form swarm was found over bare ground, the association between swarm markers and swarm molecular form was highly significant (*χ*^2^ = 56.92, d.f. = 3, *p* < 0.0001). The mixed S/M swarm (14) was found over bare ground whereas the mixed M/*An. arabiensis* swarm (33) occurred over an intersection of grassland and footpath.

### (e) Within-swarm form recognition

Overall, 455 tethered virgin females were introduced in 94 swarms of the M molecular form in the village of Sokourani during August and September of 2006 and during the same period in 2007. Of these, 47 females (10.33%) were inseminated and no significant difference in the rate of insemination was found between the forms (*χ*^2^ = 2.38, d.f. = 1, *p* = 0.122; table 2).

**Table 2.** Within-swarm discrimination using tethered females introduced into natural M swarms. *p* = 0.122.

	insemination		
	No	Yes	Total
M form	87.6%	12.4%	100%
	(212)^a^	(30)	(242)
S form	92%	8%	100%
	(196)	(17)	(213)

^a^number of mosquito females.

## 4. Discussion

In this study, we found differences in the swarming behaviour of the molecular forms of *An. gambiae* that help to explain their reproductive isolation (Tripet *et al.* 2001; Diabaté *et al.* 2006). A robust pattern of spatial segregation between swarms was found, revealing distinct form-specific mating units in sharp contrast to the mixed composition of the molecular forms indoors. Our results suggest that spatial swarm segregation in Mali is virtually complete, so it probably contributes strongly to the assortative mating between the forms. This mechanism of reproductive isolation could most easily be effective if females discriminate between swarms similarly to males. Some evidence in support of this hypothesis was obtained from analysis of 27 mating couples collected from swarms in Donéguébougou, all of which were of the same form. These results suggest that females also discriminate between swarms of their own versus the other form, although further study is needed to confirm this hypothesis. If intra-swarm recognition indeed plays a decisive role, it would be difficult to explain the sharp male segregation and the absence of ‘wrong’ females among couples collected from different swarms. Moreover, if males discriminate between swarms, and humans can use ground markers to correctly predict the form of the swarm, it is reasonable that females too can discriminate among swarms, especially because they are expected to incur a higher cost than males for cross mating. Assuming that the fitness of hybrid is reduced in nature, females are supposed to pay the highest cost in the case of cross mating, because they mate only once in their lifetime, whereas males can mate several times.

It is possible that a low rate of cross mating occurs during indoor mating, as suggested by the absence of form recognition in experiments conducted in natural huts (Dao *et al.* 2008). Indirectly, it suggests that mate recognition does not operate well outside swarms. Dao *et al.* (2008) found direct evidence for indoor mating only in an allopatric M population and proposed that in areas of sympatry, males and females of the S form depart houses before indoor mating starts. The absence of form recognition in tethered female experiments and in indoor mating provides additional evidence against the existence of within-swarm form recognition mechanisms in Mali.

In Burkina Faso, however, the absence of hybrids (Diabaté *et al.* 2006), despite the relatively high rate of mixed swarms (approx. 15%), indicates that within-swarm form recognition must operate. Although the expected frequency of mixed swarms (by chance) in Burkina Faso is substantially greater than that observed (Diabaté *et al.* 2006), we suggest that at least one additional within-swarm recognition mechanism is involved. Direct studies on the role of chemical and auditory signals will be rewarding (e.g. Gibson & Russell 2006). The repeated failure of the tethered female experiment in an area of sympatry (Donéguébougou) as opposed to the allopatric M population in Niono (only 300 km away) probably reflects yet another difference in mating behaviour between populations and suggests that the importance of mechanisms of reproductive isolation may vary geographically.

The coexistence of the Bamako and Savanna chromosomal forms within the S molecular form in Mali and not in Burkina Faso (della Torre *et al.* 2001) could contribute to this contrast in the mating behaviour between the two populations. However, because 99 per cent of the S form specimens collected from swarms in this study were of the Savanna chromosomal form, which is the only form found in Burkina Faso, this consideration cannot explain the differences. In both populations, the observed barriers operate primarily between Savanna and Mopti chromosomal forms.

Our results stress the role of ground markers as a determinant of swarm segregation in the molecular forms of *An. gambiae*. Several studies on swarming insects have found that males aggregate at certain stations (Downes 1969; Savolainen 1978; Titmus 1980; Charlwood *et al.* 2002; Yuval 2006). Consistent with our results, an allopatric S form population in Tanzania swarmed exclusively on bare ground (Marchand 1984), whereas an allopatric M form population in São Tomé used patterns of contrast as marker (Charlwood *et al.* 2002).

That only one *An. arabiensis* male was collected from swarms, despite the fact that this species comprises 10 per cent of the indoor population, suggests that *An. arabiensis* mates at specific sites not covered in our survey. Similarly, in Tanzania, no single pure swarm of *An. arabiensis* was found in an area where *An. arabiensis* and *An. gambiae* coexisted (Marchand 1984); however, swarms of *An. arabiensis* could be seen in a village where *An. arabiensis* was the only species present. The author concluded that in sympatry, *An. arabiensis* changes its swarming behaviour or mates without swarming.

The extent of reproductive isolation within *An. gambiae* has been the focus of much debate, although recent theoretical (Lehmann & Diabaté 2008 and references therein) and empirical (Turner & Hahn 2007) studies have resolved many of the issues. Our data provide evidence that swarm segregation strongly contributes to the reproductive isolation of the two forms. The question remains as to how this isolation mechanism has evolved.

Recent studies suggest that divergent selection between the forms has acted on larval traits (Diabaté *et al.* 2008). Larvae of the M form predominate in permanent larval habitats such as rice fields, whereas S larvae predominate in temporary puddles (Diabaté *et al.* 2002, 2003, 2004; della Torre *et al.* 2005). Larvae of the M form outperform S larvae in predator-rich habitats (i.e. permanent habitats), whereas S larvae outperform M larvae in the absence of predators (i.e. in temporary habitats; Diabaté *et al.* 2008). We propose that M larvae are better adapted to avoid predators than S larvae, whereas the S larvae are better adapted for competition under low predator pressure (Diabaté *et al.* 2008). Rundle and Nosil (2005), in their review on ecological speciation, stated that speciation is facilitated when genes under divergent selection cause reproductive isolation pleiotropically. The most convincing example is when reproductive isolation evolves as a direct consequence of habitat selection, assuming that individuals mate in their preferred habitat. The molecular forms of *An. gambiae* do not mate near their preferred larval habitats, and it is therefore unlikely that the genes under divergent selection in the molecular forms also cause reproductive isolation. We presume that linkage exists between genes conferring adaptive differences at the larval stage and those that influence swarming site selection. The role of divergent natural selection in speciation has been demonstrated in many species, including *Bombina* toads. Specifically, *Bombina bombina* prefers semi-permanent ponds with a higher density of aquatic predators, rather than the temporary puddles typically used by *B. variegata*. Similarly, behavioural differences in predator avoidance were reported between them in accordance with their habitat distribution (Kruuk & Gilchrist 1997). The authors presumed that the differential adaptation to cope with predation pressure led to differential choice of habitat, and indirectly to preference for alternative breeding habitats.

Although no post-mating reproductive isolation has been found in the laboratory (Diabaté *et al.* 2007), the fitness of hybrids in nature has not been tested. It is possible that hybrid inferiority contributes to reproductive barriers between the forms. In ecological speciation, post-zygotic isolation can arise when hybrids are not well adapted to either parental environment and, in effect, fall between the niches (Schluter 2001; Rundle & Nosil 2005).

Uncovering the ecological and genetic mechanisms involved in speciation is key to understanding how biological diversity is generated. Genetic differentiation between the molecular forms of *An. gambiae* and its distribution across the genome has been extensively studied, but phenotypic differences between them, the evolutionary forces that generated divergence and the mechanisms that maintain their genetic isolation have only recently been addressed (Lehmann & Diabaté 2008). Our study provides evidence that swarm spatial segregation strongly contributes to the reproductive isolation between the molecular forms of *An. gambiae* in Mali, although this does not exclude the possibility that more than one mechanism of form recognition operates across the range of the molecular forms. This study extends our understanding of the behavioural components of the speciation process and may eventually facilitate the development of new strategies for vector control.
